# H^+^-Conducting Aromatic Multiblock Copolymer and Blend Membranes and Their Application in PEM Electrolysis

**DOI:** 10.3390/polym13203467

**Published:** 2021-10-09

**Authors:** Johannes Bender, Britta Mayerhöfer, Patrick Trinke, Boris Bensmann, Richard Hanke-Rauschenbach, Katica Krajinovic, Simon Thiele, Jochen Kerres

**Affiliations:** 1 Institut für Chemische Verfahrenstechnik (ICVT), Universität Stuttgart, Boeblinger Str. 78, 70199 Stuttgart, Germany; johannes.bender@icvt.uni-stuttgart.de (J.B.); katica.krajinovic@icvt.uni-stuttgart.de (K.K.); 2 Forschungszentrum Jülich GmbH, Helmholtz Institute Erlangen-Nürnberg for Renewable Energy (IEK-11), Cauerstr. 1, 91058 Erlangen, Germany; b.mayerhoefer@fz-juelich.de (B.M.); si.thiele@fz-juelich.de (S.T.); 3 Institut für Elektrische Energiesysteme (IfES), Universität Hannover, 30167 Hannover, Germany; patrick.trinke@ifes.uni-hannover.de (P.T.); boris.bensmann@ifes.uni-hannover.de (B.B.); hanke-rauschenbach@ifes.uni-hannover.de (R.H.-R.); 4 Department Chemie- und Bioingenieurwesen, Friedrich-Alexander Universität Erlangen-Nürnberg, Immerwahrstr. 2a, 91058 Erlangen, Germany; 5 Chemical Resource Beneficiation Faculty of Natural Sciences, North-West University, Potchefstroom 2520, South Africa

**Keywords:** electrolyzer, PEM, MEA, partially fluorinated and sulfonated poly(arylene)s, multiblock-co-ionomers, acid–base blend membranes, hydrogen crossover

## Abstract

As an alternative to common perfluorosulfonic acid-based polyelectrolytes, we present the synthesis and characterization of proton exchange membranes based on two different concepts: (i) Covalently bound multiblock-co-ionomers with a nanophase-separated structure exhibit tunable properties depending on hydrophilic and hydrophobic components’ ratios. Here, the blocks were synthesized individually via step-growth polycondensation from either partially fluorinated or sulfonated aromatic monomers. (ii) Ionically crosslinked blend membranes of partially fluorinated polybenzimidazole and pyridine side-chain-modified polysulfones combine the hydrophilic component’s high proton conductivities with high mechanical stability established by the hydrophobic components. In addition to the polymer synthesis, membrane preparation, and thorough characterization of the obtained materials, hydrogen permeability is determined using linear sweep voltammetry. Furthermore, initial in situ tests in a PEM electrolysis cell show promising cell performance, which can be increased by optimizing electrodes with regard to binders for the respective membrane material.

## 1. Introduction

The production of green hydrogen through water electrolysis is currently seen as a promising option for storing electrical energy from renewable energy sources such as wind and sun and is essential for decarbonizing transport and industry. For this reason, it can be expected that water electrolysis technology will become increasingly important soon since the generated hydrogen can be used both in the mobility sector via fuel cells or as raw or auxiliary material for heavy industries (e.g., chemical or steel industry). The heart of the proton exchange membrane electrolyte (PEM) water electrolyzer (PEMWE) is the membrane electrode assembly (MEA). The oxygen evolution reaction occurs at the anode with iridium dioxide (IrO_2_) as the typical catalyst in current use. Due to the high electrode potential above 1.48 V (vs. standard hydrogen electrode), only a few materials withstand this corrosive environment. For this purpose, titanium dioxide (TiO_2_) is typically used as the catalyst support material. The hydrogen evolution reaction takes place at the cathode with platinum as the standard catalyst material. The MEA is placed between two porous current collectors. For this purpose, felts, expanded metals, or sintered materials based on titanium or made of coated stainless steel, with a thickness between 0.8 and 2 mm, are used on the anode. Porous graphite plates are usually used on the cathode side. The end of a single cell is formed on both sides by titanium or coated stainless steel plates, often with channel structures for water supply and gas removal [1,2,3]. So far, PFSA-type membranes (PFSA = perfluorosulfonic acid, e.g., Nafion^®^ [4,5,6], Aquivion^®^, Fumion^®^, Flemion^®^ or 3M^TM^) have mainly been used in PEM electrolysis [7] due to their excellent chemical stability. The thickness of the PFSA membranes typically is in the range of 20 to 200 μm. The PFSAs, however, have shortcomings such as (i) the expensive production process involving strongly toxic intermediates such as perfluorinated sulfones, (ii) doubts as to the disposal of the environmentally unfriendly F-containing ionomers after use, (iii) the still-unsolved question of PFSA recycling after use, and (iv) the high price of the PFSAs. These shortcomings have catalyzed the search for alternative membrane materials and materials for proton conduction in the electrode (ionomer). One centrally important class of materials considered as an alternative are hydrocarbon polymers [8,9] such as aromatic polyethers [10,11], polyethersulfones [12], polyetherketones [13], polyphenylphosphine oxides [14], polyphenylenes [15], polysulfones [16], and other aromatic main-chain polymers. A particular advantage of hydrocarbon ionomer membranes, compared to PFSA membranes, is the lower gas crossover, leading to a reduction in safety issues, faradaic efficiency losses and radical formation, and consequently, less membrane degradation [17,18]. There are only a few examples of the use of polymers other than PFSAs in PEMWE so far, which we briefly review here: In [19], a sulfonated poly(ethersulfone) (sPSU) membrane was investigated. With this membrane, after 35 h of potentiostatic operation at 1.8 V and 80 °C, a current density of 1.35 Acm^−2^ was reached—a performance comparable to a Nafion 115 membrane. A microbial PEMWE study was performed using a nanofiber-reinforced sulfonated poly(ether ether ketone) (sPEEK) as the membrane, with the membrane showing promising advantages over Nafion in terms of performance [20]. Another approach involved investigating sPEEK/TPA and sPSU-co-PPSS/TPA (TPA = tungstophosphoric acid) composite membranes in PEM electrolysis [21]. With these membranes, cell voltages of 1.83 V (sPSU-co-PPSS) and 1.90 V (sPEEK), at 1 Acm^−2^ and at 80 °C, were reached. The possible leaching out of TPA, which was not covalently bound to the sulfonated polymers, from the membrane matrix was, however, not investigated in this study. In [22], a partially fluorinated aromatic microblock ionomer, based on a poly(etherketone), was investigated as a proton-conducting membrane in a PEM electrolyzer. The membrane showed good proton conductivity (63 mScm^−2^) at 80 °C and 100% RH. Its performance in the PEMWE application was 1.67 V at 1 Acm^−2^ and 80 °C, which corresponds to 4 kWh Nm^−3^ of H_2_. However, compared with the current state of the art values for PFSA-based materials, these results were not impressive.

In the present study, two approaches for the development of improved proton-conducting membranes as the polymer electrolyte in PEMWE were investigated: (i) multiblock-co-ionomers based on aromatic main-chain polymers and (ii) acid–base blend membranes based on (hetero)aromatic polymers such as sulfonated polyethers, and polybenzimidazoles and pyridine-modified polyethersulfone as the basic blend components.

The advantage of multiblock-co-ionomers is their nanophase-separated morphology. While homo-ionomers are composed of one single phase, which is either highly proton-conductive with a great ion exchange capacity (IEC) and, consequently, an immense water uptake, or less conductive and, therefore, more mechanically stable. In contrast to that, multiblock-co-ionomers allow for materials with tuned properties by combining those of the individual homoblocks in a microphase separated system. Thus, materials with both high mechanical stability as well as good proton conductivity can be tailored [23,24,25,26,27].

The second proton-conducting ionomer type chosen for this study, ionically cross-linked blend ionomer membranes based on aromatic main-chain polymers, has been investigated by the Kerres group at ICVT Stuttgart for the last two decades due to their advantageous properties, such as increased proton conductivity, and improved chemical and mechanical stabilities, compared to their homo-ionomer analogs [28,29,30,31]. The acid–base blend membranes have been applied to low-[32] and intermediate temperature H_2_ fuel cells [33], perstractive alkene/alkane separation [34], and direct methanol fuel cells (DMFC) [35], as well as in H_2_-depolarized SO_2_ electrolysis [36] and redox-flow batteries [37]. Blend membranes composed of aromatic multiblock-co-ionomers and polybenzimidazole were previously applied to DMFC, leading to improved performance compared to PFSAs and pure multiblock-co-ionomers [38]. To the best of our knowledge, multiblock-co-ionomers and acid–base blend membranes have, so far, not been investigated in PEMWE, which is the goal of this study.

## 2. Materials and Methods

### 2.1. Polymer Synthesis

#### 2.1.1. Multiblock-co-Ionomers (MBI)

**Figure 1:**
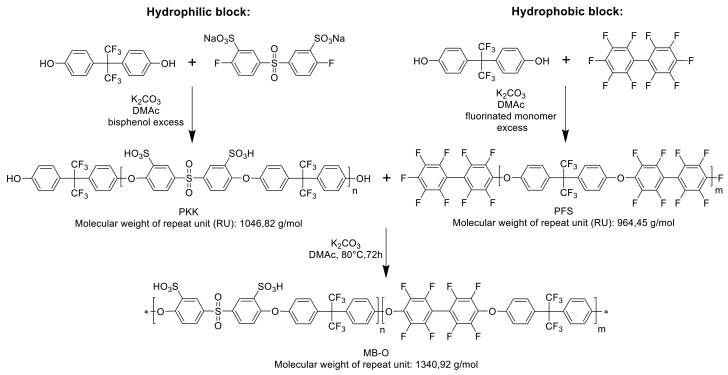
Synthesis scheme for the multiblock-co-ionomers.

The multiblock-co-ionomers were prepared in a two-step procedure: first, the hydrophilic and hydrophobic blocks were synthesized separately, followed by the blocks’ coupling in the second step, according to [20,38]. In Figure 1, the scheme for the multiblock-co-ionomer synthesis is depicted. For the synthesis of the hydrophilic block, a molar excess of the bisphenol was used, while for the preparation of the hydrophobic block, a surplus of the fluorinated monomer was utilized to yield blocks that, in the case of the hydrophilic blocks phenoxide, had the respective end groups, and, in the case of the hydrophobic blocks, had fluorinated end groups, to ensure optimally complete coupling of the blocks in the second reaction step.

In this contribution, multiblock-co-ionomers of different lengths were synthesized. Ether-bridged multiblock-co-ionomers (MBI) were synthesized from hydrophobic, partially fluorinated, and F-terminated homoblock polymer (PFS) [39] and from hydrophilic, sulfonated OH-terminated homoblock (PKK) according to previous work [23,38].

#### 2.1.2. Polymers for Three-Component Blend Membranes (^3^CBM)

**Figure 2:**
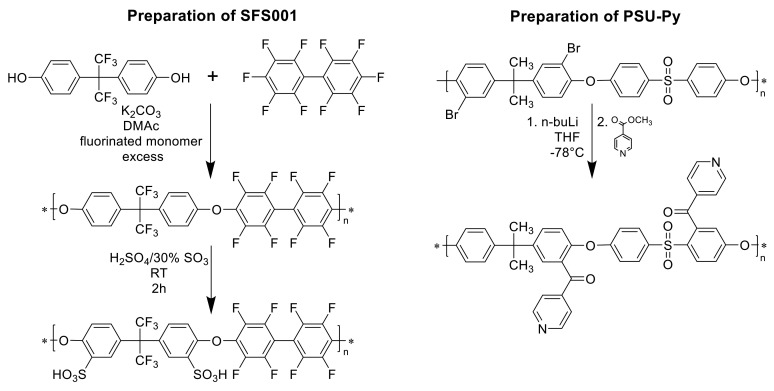
Preparation procedures for SFS001 and PSU-Py.

**Figure 3:**
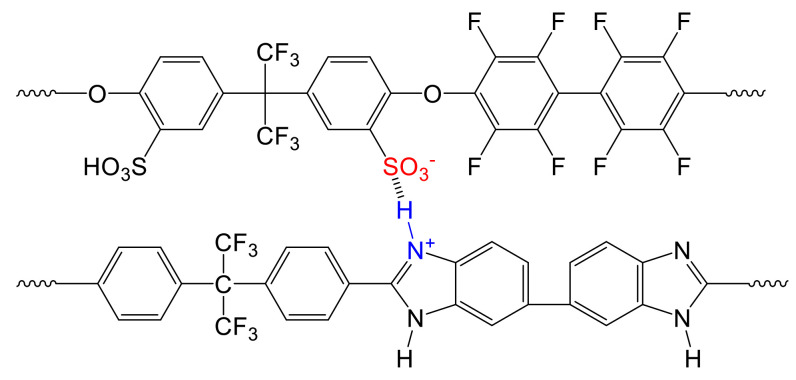
Ionic cross-linking between SFS001 and F_6_-PBI.

For the preparation of the three-component blend ionomer membranes, two types of polymers were prepared: an aromatic polyether was synthesized from bisphenol AF and decafluorobiphenyl sulfonated with H_2_SO_4_ containing 30% free SO_3_ in the second step according to [39,40]. A weakly basic pyridine-modified polyethersulfone (PSU-Py) was prepared by lithiation of PSU Udel, followed by a reaction via the isonicotinic acid methyl ester procedure described in [41]. As the second basic component of the acid–base blend membranes, the partially fluorinated polybenzimidazole F_6_-PBI was applied. The advantage of using a polybenzimidazole as a base in the acid–base blend is the resulting improved chemical and mechanical stability of the blend caused by the formation of ionic cross-link sites with the sulfonic acid groups of the sulfonated polymer [42]. The advantage of the PSU-Py is its extremely low basicity (the pK_a_ of the protonated pyridine-N calculated by ACD is 2.7+/−0.1), which leads to very “loose” ionic cross-links. Here, the protons were still somewhat mobile and expected to not be completely “captured” within the ionic cross-links. Therefore, they still contributed to proton conduction within the blend, in contrast to the ionic cross-links between PBI and the sulfonated polymer, where the proton was localized near the imidazole-N. Thus, it was drastically immobilized, which is indicated by the fact that in PBI/sulfonated polymers’ acid–base blend membranes, the calculated ion-exchange capacities were very close to the measured ones [32]. In Figure 2, the preparation procedures of the sulfonated polymer from bisphenol AF and decafluorophenyl (SFS001) and for PSU-Py are shown. Figure 3 displays the ionic cross-linking between the sulfonated polymer and the F_6_-PBI.

F_6_-PBI was purchased from Yanjin Technology, Tianjin, PR China, the hydrophilic Ionomer (SFS) was synthesized according to [39,40], and the pyridine-4-carbonyl-modified Poly(ethersulfone) Udel^®^ (PSU-Py) was synthesized as reported in previous work [41].

### 2.2. Preparation of Multiblock-co-Ionomer Membranes and Ionically Cross-Linked Blend Membranes

The multiblock-co-ionomer (3 g) was dissolved in *N*,*N*-dimethylacetamide (DMAc), or dimethylsulfoxide (DMSO) to yield a 10 wt% polymer solution. Before casting the Ionomer solution on a glass plate, it was filtered or centrifuged to remove gel particles. The solution was doctor-bladed (500–700 μm) and dried at 80 °C for 12 h, and subsequently at 130 °C for 2 h.

For blend membranes, all polymers were dissolved separately. The sulfonated polymer (2.55 g SFS001 in H^+^ form) was dissolved in *N*,*N*-dimethylacetamide to yield a 20 wt% polymer solution. Then, 1.5 equivalents of triethylamine, with reference to the polymer’s SO_3_H content, were added to the solution to neutralize the SO_3_H groups of the sulfonated polymer—without neutralization, the mixture with PBI would precipitate immediately due to the formation of a polyelectrolyte complex. Quantities of 0.15 g of F_6_-PBI and 0.3 g of PSU-Py were dissolved in DMAc (5 wt%/10 wt%). Subsequently, the basic polymer solutions were added to the SFS/Et_3_N-solution and stirred for an additional 60 min. The polymer blend solution was cast onto a glass plate (12 cm × 12 cm), and the solvent mixture was evaporated first at 80 °C for 12 h and subsequently at 130 °C under atmospheric pressure in a convection oven.

Before characterization, the membranes (MBI and ^3^CBM) were post-treated for 48 h in 10 vol% hydrochloric acid at 90 °C, followed by a post-treatment in deionized water at 90 °C for 24 h to remove excess acid.

### 2.3. Polymer and Membrane Characterization

#### 2.3.1. Water Uptake (WU) and Hydration Number (λ) (Water Molecules Per SO_3_H Group)

The dry and wet weight of the membranes was determined as described in [43]. The water uptake (*WU*) was calculated using Equation (1), and the hydration number λ (number of water molecules per SO_3_H group) was calculated by Equation (2) ($m_{H_{2}O}$ = $m_{wet}^{membrane}\;$− $\;m_{dry}^{membrane}$, $M_{H_{2}O}$ = 18 g/mol, *IEC* see Section 2.3.2) [44].

(1) $$WU=100*\;\frac{\left(m_{wet}-m_{dry}\;\right)}{m_{dry}}\;\mathrm{in}\;\%$$

(2) $$\lambda =\frac{m_{H_{2}O}}{IEC\times m_{dry}^{membrane}\times M_{H_{2}O}}$$

#### 2.3.2. Ion-Exchange Capacity (IEC)

Determination of the ion-exchange capacities (*IEC_direct_* and *IEC_total_* in mmol SO_3_H/g polymer or membrane) was performed by acid–base titration as described earlier [45]. *IEC_direct_* and *IEC_total_*, respectively, were calculated using Equations (3) and (4), shown below (Δ*V_NaOH_* = volume of consumed NaOH solution; *C_NaOH_* = concentration of the NaOH, which is 0.1 N; *m_dry_* = dry mass of membrane in H^+^ form; Δ*V_HCl_* = volume of consumed HCl solution; *C_HCl_* = concentration of the HCl, which is 0.1 N) [45]:

(3) $$IEC_{direct}=\frac{\Delta V_{NaOH}\times C_{NaOH}}{m_{dry}}$$

(4) $$IEC_{total}=\frac{\Delta V_{NaOH,excess}\times C_{NaOH}-\left[\left(\Delta V_{NaOH}\times C_{NaOH}\right)+\left(\Delta V_{HCl}\times C_{HCl}\right)\right]}{m_{dry}}$$

#### 2.3.3. Electrochemical Impedance Spectroscopy (EIS)

The proton-conductivity resistance was determined using two different EIS devices: The first EIS measurement device was the IM6 impedance workstation (Zahner Elektrik, Kronach, Germany). The conductivity measurements were performed at a frequency range from 1 kHz to 4 MHz in a 0.5 N H_2_SO_4_ solution at room temperature. Therefore, the membrane was stacked between two Nafion 117 membranes. The ohmic resistance R_ohmic_(Nafion-membrane-Nafion) was determined at the frequency, where the phase angle between current and voltage was 0°. After measurement of the three-membrane stack, R_ohmic_ of the two Nafion membranes was measured. The difference R_ohmic_(Nafion-membrane-Nafion)–R_ohmic_(Nafion-Nafion) yielded the desired impedance of the tested membranes. The second EIS device was only used for those membranes which have been also tested in situ in the electrolyzer. These measurements were carried out in a Membrane Test System (MTS740) from Scribner Associates Inc, USA. A detailed description and the technical data for the setup are given elsewhere [46]. For the sample preparation, the membrane sample in a size of 3 cm × 1 cm was compressed between two gas diffusion layers (GDLs) (SIGRACET^®^ GDL 25BC, Ion power GmbH) and attached to the platinum electrodes with conductive carbon paint. The sample compression between the electrodes was 200 PSI (or 1.38 MPa). Electrochemical impedance spectroscopy (EIS) (Impedance Analyzer 1260, Solartron Analytical) was recorded after each temperature step. The temperature of measurement was varied from 30 to 110 °C, at a constant relative humidity of 90%.

#### 2.3.4. Thermal Stability via Thermogravimetry Coupled with a Fourier–Transform Infrared Spectrometer (TGA–FTIR)

The thermal stability of the synthesized polymers and membranes was determined via thermogravimetric analysis (TGA). The samples were heated in the TGA cell of a Netzsch STA 449C or STA 449 F3 Jupiter in an oxygen-rich atmosphere (60–75% O_2_, 40–25% N_2_) in an aluminum oxide crucible at a constant heating rate (20 K/min) to a previously defined maximum temperature T_max_ (600 °C). This instrument was coupled via a heated transfer line to an FT-IR spectrometer (Nicolet 6700 Thermofisher or Bruker Perseus FT-IR), with which the series of FTIR spectra of decomposition gases was measured. Every FTIR spectrum could be assigned to a particular temperature at which it was recorded. T_SO__3__H-onset_ was identified via the asymmetrical stretching vibration of the S=O bond (1352–1342 cm^−1^), indicating the onset of SO_3_H group splitting-off, and T_CO-onset_ via the valence vibration of the C=O bond (2144 cm^−1^), indicating the onset of backbone degradation [47].

#### 2.3.5. Size Exclusion Chromatography (SEC)

Size exclusion chromatography (SEC) or gel permeation chromatography (GPC) allows the determination of the molecular weight distribution (MWD, M_n,_ and M_w_) of a polymer by its fractionation based on hydrodynamic volume of the macromolecular chains of the respective chain lengths. The MWD allows averaging for M_n_ and M_w_ with M_n_ < M_w_. The width of an MWD is called polydispersity (PD) and is calculated from the quotient M_w_/M_n_. Calibration was performed with polystyrene standard (PSS ReadyCal Kit). *N,N*-dimethylacetamide (DMAc), to which 5.0 g per L lithium bromide (LiBr) was added, which was used as the mobile phase (eluent). The samples were measured using an Agilent Technologies 1200 Series GPC system. A differential refractometer (Shodex RI 71), a viscometer (PSS ETA-2010), and a multi-angle light scattering (MALLS) photometer (PSS SLD 7000) served as detectors. The stationary phase consisted of separation columns from Polymer Standards Service GmbH (PSS), namely a column combination of different pore sizes (PSS PFG, 5–7 μm, 300 Å; PSS PFG 5–7 μm, 1000 Å).

#### 2.3.6. Stress–Strain Measurements of Selected Membranes

Mechanical properties of the membranes were measured at room temperature by using an EZTest Compact Tabletop Testing Machine (SHIMADZU Corporation, Kyoto, Japan). Prior to the measurement, all membranes were cut into rectangular shapes with areas of 1 cm × 5 cm. During measurement, every membrane was held moderately at both ends by using metal clamps. Membranes were stretched parallel to the machine direction with a constant speed of 5 mm/min.

#### 2.3.7. Determination of H_2_ Permeability of the Membranes

The hydrogen permeability of the membranes was measured via improved linear sweep voltammetry (LSV) according to Pei et al. [48]. In using this technique, we used 5 different sweep rates (SR) of 10, 8, 6, 4 and 2 mV s^−1^ in a voltage range between 0.05 and 0.6 V. Due to the capacitive effects, the measured currents were strongly affected by the SR. Therefore, the measurements for the 5 different SRs were used to extrapolate the theoretical current at a SR of 0 mV s^−1^, which could be assumed to be free of capacitive effects. Additionally, for thin membranes, it was important not to neglect the share of the ohmic short currents through the membranes. Therefore, the resulting voltammetry curves (*i* vs. *U*) at an infinitely small SR of 0 mV s^−1^ were also linear extrapolated to gain the y-axis intercept that was equivalent to the hydrogen crossover current density $i_{H_{2}}^{\mathrm{cross}}$, which was assumed to be free of ohmic short currents.

By assuming that the main crossover mechanism was the diffusive transport of dissolved hydrogen through the aqueous phase of the membrane [49] and that this transport can be described by Fick’s first law of diffusion [7,49], then the permeability coefficient, according to hydrogen $K_{P,{\;H}_{2}}$, for the different membrane materials could be calculated by Equation (2). Furthermore, it was assumed that the hydrogen pressure on the anode side could be neglected and that the cathode hydrogen gas at the membrane was fully saturated with vapor, since the gas was fully vapor-saturated at the cell outlet. Consequently, the difference of the partial hydrogen pressure across the membrane $\Delta p_{H_{2}}$ could be calculated by subtracting the saturation vapor pressure from the applied cathode pressure of 1 bar.

(5) $$K_{P,{\;H}_{2}}=\frac{i_{H_{2}}^{\mathrm{cross}}\delta^{\mathrm{mem}}}{2F\Delta p_{H_{2}}}$$

The hydrogen crossover tests for the synthesized and reference membranes were conducted in electrolysis cells from balticFuelCells with active areas of 4 and 25 cm². The cells were placed in a Greenlight E100 test station [50]. The heated deionized water feed on the anode side was set to 50 mL min^−1^ at atmospheric pressure conditions. The cathode side was supplied by dry hydrogen gas with a nominal flow rate of 0.05 m³ min^−1^. For the LSV measurements, a ModuLab XM ECS (Solartron analytical) were used, which was equipped with an XM FRA 1 MHz, an XM PSTAT AUX and an XM BOOSTER 2A modules.

Electrodes for the crossover measurements via LSV were prepared by spray coating an ink with 1 wt% solid content, composed of 20 wt% Nafion D520 and 80% platinum on carbon catalyst (HiSPEC 9100, Johnson Matthey) in a 1:4 2-propanol/water mixture, onto H_24_C_5_ gas diffusion layers (Freudenberg) [51]. The Pt loading was 0.2 mg cm^−2^.

### 2.4. PEM Water Electrolysis Experiments

#### 2.4.1. Fabrication of Membrane Electrode Assemblies (MEAs)

All MEAs shown in this work were based on free-standing membranes clamped between the same type of porous transport electrodes with an active area of 5 cm^2^. For the anode, porous transport electrodes with an IrO_2_ (Premion, Alfa Aesar) loading of 1.5 mg cm^−2^ and 9 wt% Nafion D520 (Chemours) binder were prepared, according to a spray coating procedure outlined by Bühler et al., on grade 1 titanium fiber sinter substrates (2GDL40-1.0, Bekaert) using a SonoTek ExactaCoat device with an AccuMist spray nozzle [52]. The cathodes were H_24_C_5_ gas diffusion layers (Freudenberg) with 0.5 g cm^−2^ Pt loading fabricated in the same fashion as described above.

Nafion 212 membranes (Chemours) were soaked in deionized water for 30 min before cell assembly. The Multiblock-co-Ionomer and blend membranes were used as synthesized. MEA compression was set to approximately 28% using PTFE gaskets (HighTechFlon) with a thickness of 0.18 mm and 1.00 mm at cathode and anode, respectively.

#### 2.4.2. Electrolysis Tests

All electrolysis tests were conducted on a commercial test bench (600-ETS, Scribner Associates) using the corresponding test cell fixture with meander shaped flow pattern to supply 40 mL min^−1^ deionized water and 100 mL min^−1^ nitrogen to the anode and cathode, respectively. All electrolysis tests were performed in the same fashion, equilibrating the cell at 80 °C for 1 h before the break-in period of 1 h at 1.8 V. Afterwards, three galvanostatic polarization curves were recorded at ambient pressure, of which the first one always differed from the second and the third one, whereas the latter ones were the same. For this reason, the third polarization curve of every experiment is shown here.

## 3. Results

The preparation and characterization of the aromatic polymer blocks, multiblock-co-ionomers and ternary blend membranes is described in Section 3.1. Afterwards, in Section 3.2 and Section 3.3, the results of the hydrogen crossover and polarization measurements of the synthesized membranes are shown and discussed in comparison to Nafion.

### 3.1. Preparation and Characterization of Ionomers and Ionomer Blend Membranes

#### 3.1.1. Preparation and Characterization of the Aromatic Polymer Blocks

As indicated in Figure 1, the hydrophilic blocks were synthesized by polycondensation of sodium 5,5’-sulfonylbis(2-fluorobenzenesulfonate) and bisphenol AF, and the hydrophobic blocks were synthesized by the reaction of decafluorobiphenyl and bisphenol AF. The chain length n (n = number of repeat units) of the blocks was thereby modified by variation of the molar ratio r between the bisphenoxy and bisfluoro monomer, respectively. In Table 1, the characterization results of the different synthesized hydrophilic blocks are listed; Table 2 includes those of the hydrophobic blocks. All synthesized homopolymers with similar molecular weights (M_n_) were merged and assigned to different categories such as short, middle, and long (chain).

**Figure 4:**
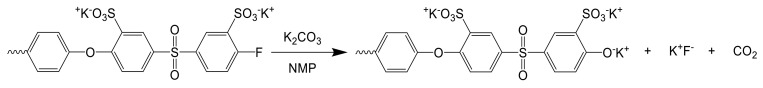
Hydrolytic splitting-off of F^-^ ions as a side reaction.

Suppose one compares the theoretically calculated chain lengths of the hydrophobic and hydrophilic blocks with the experimentally determined ones in both tables. In that case, it is noticeable that the hydrophilic chains’ actual chain length is considerably less than that calculated from the molar ratio of the monomers, whereas this is not the case for the hydrophobic blocks. This finding can be explained as follows [48]: during step polycondensation of the hydrophilic blocks, a substitution of a part of the F from the fluorinated oligomer end groups by O^−^K^+^ groups can take place by means of a nucleophilic attack of the basic K_2_CO_3_ catalyst, which terminates the polycondensation reaction (see Figure 4).

**Figure 5:**
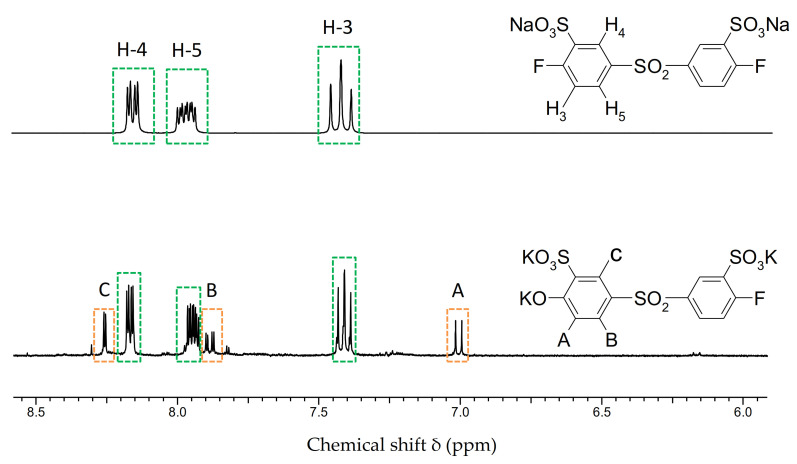
^1^H NMR spectra in DMSO-d_6_ of SDFDPS before (top) and after (bottom) the hydrolytic stability test [48].

With the aim of checking the validity of this assumption, a study was carried out on the hydrolytic stability of fluorine atoms on aryl monomers [53]. The influence of inductive and mesomeric effects on the hydrolytic stability of F atoms was demonstrated on the monomers di-sodium-4,4’-difluorodiphenylsulfone-3,3’-disulfonate (SDFDPS), 4,4’-difluorodiphenylsulfone (DFDPS) and Decafluorobiphenyl (DFBP). The monomers were each exposed to a 12-fold excess of K_2_CO_3_ in NMP for 24 h at 130 °C. It was expected that the hydrolysis would be favored by electron-withdrawing acceptor substituents (-SO_2_-, -SO_3_K) on the aromatic ring, since these reduce the electron density of the ring, which favors the attack by nucleophiles. Proof was furnished that hydrolytic cleavage of F ions only occurs in SDFDPS. With DFBP, only electron-withdrawing, inductive (–I) effects worked, so no F splitting-off was observed. Inductive (I) effects are generally weaker than mesomeric (M) effects. In DFDPS, the mesomeric (–M) effect of the sulfone bridge (-SO_2_-) acts in addition to the –I effect of the F atoms. However, only the additional –M effect of the potassium sulfonate group (SO_3_K) of the SDFDPS leads to the elimination of the terminal F atoms. Figure 5 shows the ^1^H NMR spectra of the monomer SDFDPS before and after the hydrolytic stability test. The additional signals in the ^1^H-NMR spectra (A, B and C) of the reaction product after the test are due to the di-sodium-4,4’-dihydroxydiphenylsulfone-3,3’ resulting from the nucleophilic substitution of F by OK as the side reaction.

#### 3.1.2. Preparation and Characterization of the Multiblock-co-Ionomers

The previously described hydrophilic, OH-terminated (PKK), and hydrophobic, F-terminated (PFS) blocks were coupled together via a polycondensation reaction at an equimolar ratio (r = 1). In Table 3, the characterization results of the multiblock-co-ionomers are listed. The highest molecular weights were achieved by combining long hydrophilic and hydrophobic blocks. Gelation was observed using more than three molar equivalence of potassium carbonate or at long reaction times (>24 h) at 80 °C. The degradation of the ionomers increased with higher temperatures. It turned out that the polycondensation reaction between the homoblocks ended in equilibrium with 3 to 4 coupled blocks, and all the attempts to increase them failed due to degradation or gelation. Nevertheless, membranes prepared from MBI-LL, MBI-LS, and MBI-MS showed the best film formation properties. All the others were brittle (Table 4). As expected, the H^+^ impedance as well as swelling decreased with longer hydrophobic blocks.

#### 3.1.3. Preparation and Characterization of the Polymers Used for the Preparation of Ternary Blend Membranes

Table 5 provides some characterization data of the polymers used as components for the ternary blend membranes.

#### 3.1.4. Characterization Data of the Ternary Blend Membranes

In Table 6, the synthesis and characterization data of the ternary blend membranes, which were obtained by blending of SFS001, PSU-Py, and F_6_PBI, are listed.

**Figure 6:**
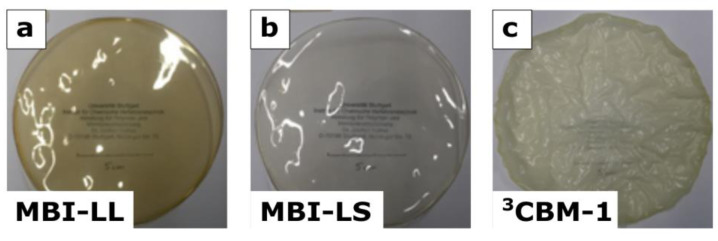
Photographs of the multiblock-co-ionomer membranes: (**a**)—MBI-LL; (**b**)—MBI-LS; (**c**)—the ternary blend membrane ^3^CBM-1.

All the membranes were transparent, indicating miscibility of the blend components, which can be explained by the interactions present in the blend membranes, such as hydrogen bridges and acid–base cross-links (electrostatic interactions) between the blend components (see Figure 6). The membranes listed in Table 4 and Table 6 were characterized in terms of H_2_ permeability and performance in a PEMWE device.

### 3.2. Selected Characterization Results of Those Membranes Which Were Applied to Electrolysis

For those of the aromatic membranes that were applied to electrolysis experiments (the block-co-ionomer membrane MBI-MS and the blend membrane ^3^CBM-2), in-depth characterization was performed. The detailed characterization included stress–strain measurement results, TGA-FTIR coupling experiment results, and the determination of proton conductivity in dependence of temperature via the impedance spectroscopy in a Scribner EIS device. Below, the results of these characterizations are presented and discussed.

#### 3.2.1. Stress–Strain Measurement Results

**Figure 7:**
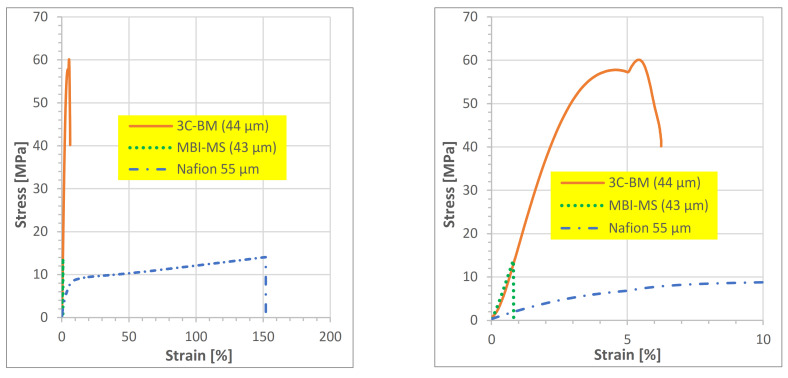
Stress–strain measurements of the membranes MBI-MS, ^3^CBM-2 and, for comparison, Nafion 212. Left: complete stress–strain curves of all membranes; right: extract of the stress–strain curves for the strain range of 0 to 10%.

In Figure 7, the stress–strain curves of the membranes MBI-MS, ^3^CBM-2 and, for comparison, Nafion 212, are depicted. In Figure 7, the difference in tensile properties between the aromatic polymer membranes and Nafion is clearly indicated: the aromatic polymer membranes show a higher tensile stress, but much lower tensile strain than Nafion, which confirms stress strain measurement results of other studies, such as [54], and which can be explained by the stiff nature of aromatic ionomer macromolecules, compared to PFSAs, which consist of much more flexible perfluoroalkyl backbones and flexible perfluoroether side chains. Furthermore, when comparing the stress–strain curve of the 3C-BM blend membrane with that of the block co-ionomer membrane, one can clearly see the higher flexibility and mechanical strength of the blend membrane; this can be traced back to the ionic cross-links between the acidic and basic polymeric components of this acid–base blend membrane, which give it greater mechanical flexibility than the pure aromatic block co-ionomer membrane, as has already been determined in earlier studies by the Kerres group [30,31].

#### 3.2.2. TGA-FTIR Coupling Results

**Figure 8:**
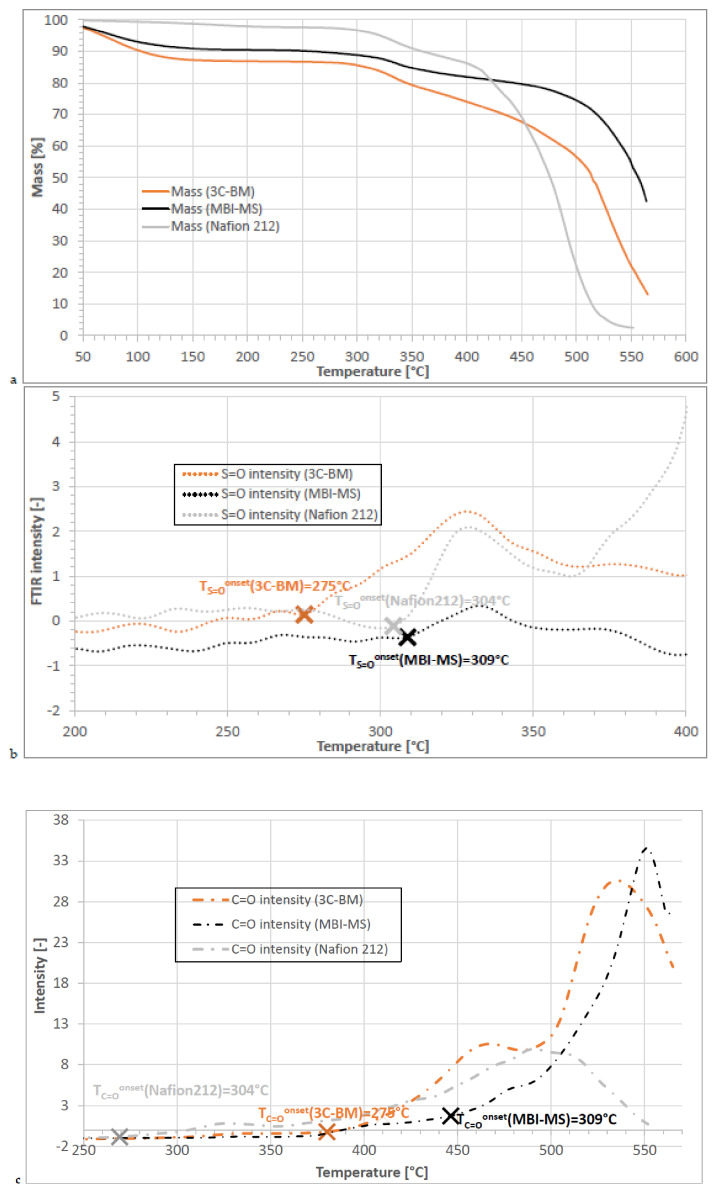
TGA-FTIR coupling results of the three membranes 3C-BM, MBI-MS and Nafion 212; (**a**): TGA traces in dependence of temperature; (**b**): S=O FTIR intensity in dependence of T; (**c**): C=O FTIR intensity in dependence of T.

In this section, it will be shown, through the examples of the membranes MBI-MS, 3C-BM and Nafion 212 (those membranes that were investigated in the PEM electrolyzer cell), how the start temperatures of SO_3_H group splitting-off (indicated by the first appearance of the S=O vibration in the FTIR gas cell coupled to the TGA cell; for details, see Section 2.3.4) and the start temperature of backbone degradation (indicated by the first appearance of the C=O vibration in the FTIR gas cell; for details, see also Section 2.3.4), were determined. In Figure 8a, the TGA traces of the three membranes are depicted, while in Figure 8b, the curves for S=O vibration intensities of the three membranes in dependence of T are shown, and in Figure 8c, their C=O vibration intensities, respectively, in dependence of T, are shown. From Figure 8, it can be seen that the aromatic 3C-BM and MBI-MS membranes show thermal stabilities comparable to Nafion 212. In the temperature region up to 280–300 °C, only residual evaporation of water and solvent are responsible for the weight loss, as could be ascertained by the TGA-FTIR coupling experiments [47]. From Figure 8b, which displays the S=O vibration intensity in dependence of T, the onset temperatures of SO_3_H splitting-off of the three membranes are marked with an X, their values being in the same range as observed in earlier studies for comparable membranes (for membranes being comparable to 3C-BM, see study [55], and for membranes being comparable to MBI-MS, see study [23]). In Figure 8c, which presents the C=O vibration intensities of the three membranes in dependence of T, in which, again, the onset temperatures of C=O formation (identical with the start temperature of backbone degradation [47]) are marked with an X, one can see that (1) the onset of polymer backbone degradation of the 3C-BM blend membrane is at a lower temperature than at the MBI-MS membrane, which can be traced back to the PSU-py blend membrane component whose C=O formation onset temperature was determined to be 310 °C (see Table 5), and (2) that the backbone degradation of Nafion 212 starts even at a slightly lower temperature than the splitting-off of its SO_3_H groups, which can be explained by the radical degradation process of Nafion-type membranes, investigated in a quantum mechanics mechanistic study, involving the simultaneous splitting-off of the SO_3_H group and degradation of the side chains under the formation of different splitting products [56]. From the TGA-FTIR coupling experiments, it can be concluded that all the membranes investigated in this study show excellent thermal stabilities with thermal degradation temperatures being much higher than the common PEMWE electrolyzer operation temperatures of 60–90 °C.

#### 3.2.3. Electrochemical Impedance in Dependence of Temperature

**Figure 9:**
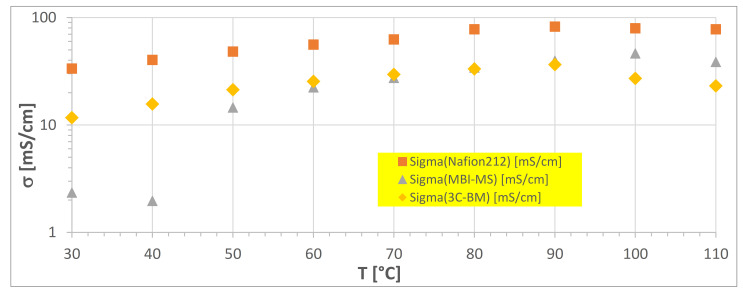
EIS measurement results of the membranes ^3^C-BM, MBI-MS and Nafion212 in dependence of temperature.

Electrochemical impedance spectroscopy investigation was performed with the three membranes in dependence of temperature from 30 to 110 °C, at a humidification rate of 90%, with a Scribner device. The results of the EIS measurements are displayed in Figure 9.

From Figure 9, it can be seen that the conductivity values are, in the case of the two polyaromatic membranes, significantly lower than that of Nafion 212, although they show an in situ electrolysis performance comparable to that of the Nafion 212. This discrepancy can be explained as follows: Measurements in the Scribner EIS device are performed in water vapor (humidification degree 90%), while in in situ electrolysis cells, the membranes are in contact with liquid water. Furthermore, the difference in water uptake of an ionomer membrane when being in contact with liquid water (higher water uptake) or water vapor (lower water uptake) has been known for many years as Schroeder’s Paradox (an explanation for this behavior is given by Freger in [57]). Moreover, the H^+^ conductivity of sulfonated ionomer membranes is dependent on their water content: the higher the water content is, the higher the H^+^ conductivity [58]. When comparing the conductivity values measured with the Scribner device (water vapor) with those measured with the Zahner device (0.5 N aqueous H_2_SO_4_ solution), it is clearly seen that the conductivity values from the Zahner cell are significantly higher than those measured with Scribner. Of course, it must also be noted that, since the Zahner measurements were carried out in 0.5 N H_2_SO_4_ solution, the acid solution has an additional effect on the conductivity, because the contact resistance between membrane and electrodes is much lower with EIS measurements in an acid solution than is the case with measurements in water, as observed in an earlier study [41].

### 3.3. Hydrogen Permeability Measurements

**Figure 10:**
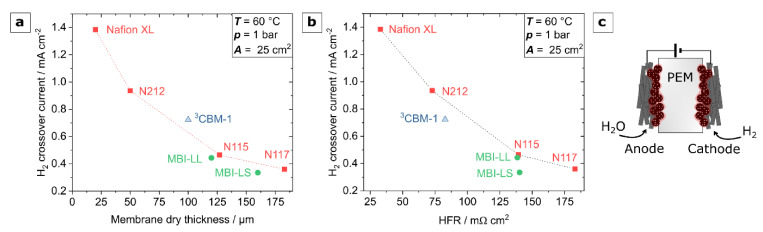
Results of LSV experiments performed under realistic electrolysis conditions. (**a**)—hydrogen crossover current as a function of membrane dry thickness; (**b**)—hydrogen crossover current as a function of HFR; (**c**)—schematic of the test setup with membrane clamped between Pt/C based gas diffusion electrodes and a water supply from the anode.

For hydrogen safety and optimum efficiency of a water electrolyzer, hydrogen permeability through the membrane material is crucial. Therefore, we investigated this parameter in LSV measurements under electrolysis conditions with a water feed from the anode, as depicted in Figure 10c. The permeability results, as a function of the membrane’s dry thickness and its HFR, compared to a series of commercial Nafion membranes, are depicted in Figure 10a,b. Figure 10a shows that the multiblock-co-ionomers MBI-LL and MBI-LS were superior to the Nafion samples for a given membrane thickness, whereas the ternary blend membrane revealed a slightly worse performance. However, considering the implementation of these membranes in a real electrolyzer, the HFR of the membrane is the performance-determining parameter, which is shown in Figure 10b instead of the membrane thickness. With regard to the hydrogen crossover to resistance ratio, all samples performed better than the Nafion reference. This improved crossover to resistance ratio in comparison to Nafion was also measured by Albert et al. [59] with radiation-grafted membranes and by Klose et al. [60] with a hydrocarbon membrane. The radiation-grafted membranes showed comparable beneficial results such as the membranes of this work [59]. However, the hydrocarbon membrane revealed an even better crossover to resistance ratio, mainly caused by the very good proton conductivity, i.e., small HFR [60]. In a recent work conducted by Chae et al. [61], multiblock copolymers with highly acidic fluorinated-hydrophilic domains also show lower gas permeabilities as compared to Nafion. Additionally, there are also other PFSA membranes, such as Aquivion [62] and Fumapem [50], which show lower diffusive hydrogen permeability coefficients in comparison to Nafion, but for these membranes, the crossover to resistance ration was not yet measured.

### 3.4. Water Electrolysis Polarization Behavior

**Figure 11:**
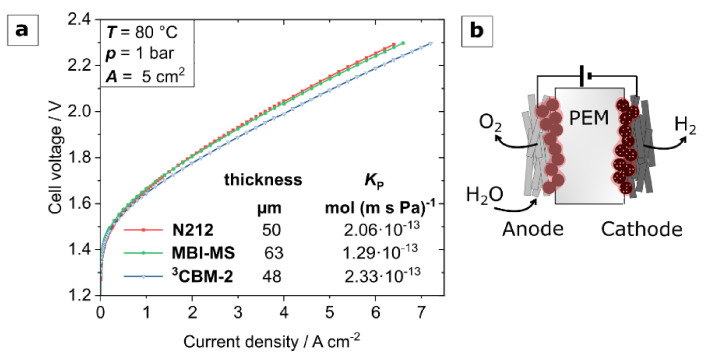
(**a**)—Performance of MBI-MS and ³CBM-2 membranes compared to Nafion 212 in water electrolysis operation at 80 °C; (**b**)—schematic of the MEA design composed of a free-standing membrane and porous transport electrodes for the anode and cathode, respectively.

As the conductivity of both MBI-MS and the ternary blend membrane (³CBM), and their hydrogen permeability from LSV measurements, appeared very encouraging, their performance in a water electrolysis MEA was investigated. A comparison of their polarization data to a Nafion 212 membrane reference is depicted in Figure 11a. It must be noted that electrodes and MEA operating parameters in this work were optimized for a Nafion-based PEMWE-system. Even for these non-optimum conditions, a 63 µm thick MBI-MS membrane’s performance was similar to that of a 50 µm thick Nafion membrane. This performance is in a comparable range with recently reported hydrophilic-hydrophobic multiblock copolymers by Chae et al. [61], which are also found to exceed a Nafion 212 membrane when operated at 90 °C with PFSA-based electrodes [61]. Earlier studies on microblock ionomers [22,63] show inferior performance values. However, due to the variation in MEA fabrication method, membrane thickness and operating temperature, a valid comparison is not possible.

The 48 µm thick ternary blend membrane could even outperform the Nafion reference, reaching a current density of 4.1 Acm^−2^ at a cell voltage of 2.0 V. These preliminary results are very promising. However, optimization of the MEA to the respective membranes, including more compatible electrode binders, e.g., containing the same aromatic ionomers as in the membrane, and a thorough investigation of the hydrogen crossover and durability, has to be pursued in further studies. In addition to the polarization behavior, the hydrogen permeability coefficients of the investigated membranes are stated in Figure 11a. The hydrogen crossover was measured via LSV with pieces of the same membrane as used in the polarization tests, also at 80 °C. The given permeability coefficients are related to the dry membrane thickness and the same trend as in Figure 10a can be seen. Regarding the crossover flux related to the membrane thickness, the MBI sample is superior to Nafion and the ³CBM membranes are slightly inferior. However, therefore the polarization behavior is better and, consequently, thicker membranes with less hydrogen crossover fluxes could be used, since the crossover to resistance ratio is better of both membranes in comparison to Nafion (cf Figure 10b).

## 4. Conclusions

In the present study, partially fluorinated hydrocarbon membranes, based on arylene main-chain multiblock-co-ionomers (MBIs) and ternary arylene main-chain acid–base blend membranes, were synthesized and characterized. For the synthesis of the block-co-ionomers, both hydrophobic and hydrophilic blocks were synthesized separately by nucleophilic displacement polycondensation and, subsequently, were coupled to multiblock-co-ionomers, also by polycondensation. As hydrophilic blocks, aromatic sulfonated oligomers with OH end groups were prepared. As hydrophobic blocks, partially fluorinated aromatic oligomers with F end groups were synthesized. By applying different molar mixing ratios between the bisphenol and the fluorinated aromatic compounds, different block lengths were obtained. Interestingly, for the hydrophilic blocks, in all cases, lower molecular weights than calculated were found, which can be explained by nucleophilic substitution of a part of the end-Fs of the disulfonated 4,4’-difluorodiphenylsulfone units by OH^−^ ions formed by hydrolysis of the K_2_CO_3_ base. The mechanically stable MBI membranes showed good proton conductivities between 50 and 100 mS/cm. The ternary acid–base blend membranes, which were also prepared, showed good proton conductivities as well, being in the range between 56 and 138 mS/cm.

The best two synthesized polymers/polymer blends were tested in a water electrolysis cell. The membranes were each placed between identical porous electrodes within a water electrolysis cell. The electrochemical performance of both applied membranes was slightly better in comparison to a commercial N212 membrane. Additionally, the hydrogen permeability coefficient of the MBI-MS membrane was also smaller than that of the reference N212 membrane, which was determined by hydrogen crossover measurements via improved linear sweep voltammetry. The permeability value of ternary blend membrane was comparable to that of the Nafion 212 membrane; however, the ratio of crossover flux to membrane resistance was even better for both investigated membranes in comparison to Nafion. These are very promising results, since the porous electrodes were not optimized for the investigated partially fluorinated ionomer membranes. The good proton conductivities of the investigated arylene main-chain block-co-ionomer membranes can be traced back to the nanophase-separation between hydrophilic and hydrophobic blocks in the block-co-ionomers, as has been proven in the past by other studies, leading to high local concentrations of H^+^-conductive SO_3_H groups that form proton-conductive channels within the membrane. The observed lower H_2_ crossover of the MBI membranes is due to the hydrophobic nanophase of the MBIs, which impedes H_2_ permeation.

For further investigations, it is important to characterize the long-term stability of these membranes, especially under real electrolysis conditions, since this is one of the most important issues for technically relevant membranes. Moreover, the MEAs have to be optimized for the application of electrolysis, in which the electrodes, in particular, have to be optimized using the same ionomer in the electrode as in the membrane, which is not trivial and will be the topic of ongoing studies with the ionomer membrane types presented here. For future polymer and membrane development, it is also intended to prepare arylene main-chain polymers that do not contain electron-rich ether or thioether bridging groups, which can be attacked by electrophilic radicals formed in the electrochemical electrode reactions, leading to chain scission.

## Figures and Tables

**Table 1:** Characterization results of the hydrophilic blocks (average values for n_calc_, M_w_, M_n_, IEC, Yield, and T_onset_).

Name	Ratio r ^(a)^	n_calc_^(b)^	M_w_ kDa	M_n_ kDa	PDI ^(c)^	n_GPC_^(d)^	IEC_dir_ meq/g	IEC_tot_ meq/g	Yield %	T_SO_3_H-onset_ ^(e)^ °C
PKK long	0.993	143	18.8	10.2	1.8	14	0.42	0.85	70	290
PKK middle	0.972	36	12.3	8.3	1.5	12	1.41	1.77	42	280
PKK short	0.955	22	6.6	5.1	1.3	7	1.08	1.47	39	270

^(a)^ r = N_a_/N_b_, where N_a_ = molar amount of bisphenolate and N_b_ = molar amount of bisfluoro monomer; ^(b)^ chain length (number of repeat units), calculated from r [39]; ^(c)^ PDI = M_w_/M_n_; ^(d)^ chain length (number of repeat units), determined via GPC from M_n_; ^(e)^ determined via TGA-FTIR coupling, a method described in [47].

**Table 2:** Characterization results of the hydrophobic blocks (average values for n_calc_, M_w_, M_n_, IEC, Yield and T_onset_).

Name	Ratio r ^(a)^	n_calc_^(b)^	M_w_ kDa	M_n_ kDa	PDI ^(c)^	n_GPC_^(d)^	Yield %	T_SO_3_H-onset_ ^(e)^ °C
PFS long	0.936	15	24.7	10.1	2.4	16	81	450
PFS short	0.818	5	9.0	4.7	2.0	8	85	420

^(a)^ r = N_a_/N_b_, where N_a_ = molar amount of bisphenolate and N_b_ = molar amount of bisfluoro monomer; ^(b)^ chain length (number of repeat units), calculated from r [39]; ^(c)^ PDI = M_w_/M_n_; ^(d)^ chain length (number of repeat units), determined via GPC; ^(e)^ determined via TGA-FTIR coupling, a method described in [47].

**Table 3:** Characterization data of the synthesized multiblock-co-ionomers (MBI) (average values for M_w_, M_n_, IEC and T_onset_).

Name/Type	Composition PKK + PFS ^(a)^	M_w_ kDa	M_n_ kDa	PDI ^(b)^	n_GPC_ ^(c)^ blocks	IEC_dir_ meq/g	IEC_tot_ meq/g	T_SO_3_H-onset_ ^(d)^ °C	T_CO onset_ °C ^(d)^
MBI-LL	Long + Long	104.3	41.3	2.5	4	0.40	0.62	291	409
MBI-LS	Long + Short	67.7	25.3	2.7	3	0.88	0.99	270	497
MBI-ML	Middle + Long	79.9	21.9	3.6	2	0.65	0.92	-	-
MBI-MS	Middle + Short	63.8	22.2	2.9	3	0.57	0.90	303	475
MBI-SL	Short + Long	gel	gel	-	-	0.64	1.01	-	-
MBI-SS	Short + Short	110.6	19.5	5.7	4	0.49	0.80	287	457

^(a)^ Composition of **M**ulti**B**lock-co-**I**onomers (MBI); ^(b)^ PDI = M_w_/M_n_; ^(c)^ chain length (number of blocks), determined via GPC and calculated from [2*M_n_ (MBI)/M_n_ (PKK+PFS)]; ^(d)^ determined via TGA-FTIR coupling, a method described in [47].

**Table 4:** Characterization data of the synthesized multiblock-co-ionomers membranes (MBI) (average values for thickness, IEC, T_onset_ and impedance).

Type	Thickness μm ^(a)^	IEC_dir_ meq/g	IEC_tot_ meq/g	T_SO_3_H onset_ °C ^(b)^	T_CO onset_ °C ^(b)^	WU 25°/90 °C % ^(c)^	λ 25°/90°^(d)^	T_g_ [°C] ^(e)^	Specific Imped. Ω*cm ^(f)^	Conductivity mS/cm ^(g)^
MBI-LL	105	0.52	0.66	323	471	20.7/23.2	17.4/19.5	178.4	17.1	58.5
MBI-LS	126	0.98	1.1	342	477	19.3/25.3	9.7/12.8	191.8	13.3	75.2
MBI-ML	Brittle	n. d.	n. d.	n. d.	n. d.	n. d.	n. d.	n. d.	n. d.	n. d.
MBI-MS	82	1.1	1.34	309	446	48.1/60.5	19.9/25.1	-	9.8	102.0
MBI-SL	Brittle	n. d.	n. d.	n. d.	n. d.	n. d.	n. d.	n. d.	n. d.	n. d.
MBI-SS	Brittle	n. d.	n. d.	n. d.	n. d.	n. d.	n. d.	n. d.	n. d.	n. d.

^(a)^ Average thicknesses; ^(b)^ determined via TGA-FTIR coupling, a method described in [47]; ^(c)^ WU = water uptake at 25 and 90 °C; ^(d)^ λ = water molecules per SO_3_H group, calculated according to Equation (2) (see Section 2.3.1); ^(e)^ determined via DSC; ^(f)^ impedance determined at r.t. in 0.5 N H_2_SO_4_ solution using the Zahner EIS device; ^(g)^ calculated from 1000/Ω*cm.

**Table 5:** Characterization data of the (synthesized) blend membrane components.

Name	M_w_ kDa	M_n_ kDa	PDI ^(b)^	n_GPC_ ^(c)^	IEC_tot_ meq/g	DS ^(e)^	T_SO_3_H onset_ °C ^(f)^	T_CO onset_ °C ^(f)^
SFS001	211.5 ^(a)^	67.5 ^(a)^	3.1	85	2.31	-	308	451
PSU-py	38.3	14.3	2.7	21	-	2.13	-	310
F6-PBI	847.3	66.1	12.8	123	3.74 ^(d)^	-	-	500

^(a)^ Average molecular weight distributions; ^(b)^ PDI = M_w_/M_n;_^(c)^ chain length (number of repeat units), determined via GPC (calculated from M_n_); ^(d)^ calculated from repeat unit. ^(e)^ calculated from elemental analysis results; ^(f)^ determined via TGA-FTIR coupling, a method described in [47].

**Table 6:** Synthesis and characterization data of the ternary blend membranes from SFS001, PSU-Py and F_6_-PBI.

Name	Ratio SFS/PSU-py/ F6PBI ^(a)^	Thickness μm ^(b)^	IEC_dir_ meq/g	IEC_tot_ meq/g	T_SO_3_H onset_ °C ^(c)^	T_CO onset_ °C ^(c)^	WU 25°/90 °C % ^(d)^	λ 25°/90° ^(e)^	T_g_ °C ^(f)^	Spec. Imped. Ω*cm ^(g)^	Cond. mS/cm ^(h)^
³C-BM-1	85:12:3	100	1.38	1.71	310	425	76/130	24.7/42.2	314	17.8	56.3
³C-BM-2	85:10:5	25	1.19	1.79	275	380	61/108	18.9/33.5	-	7.3	137.7

^(a)^ Weight ratio between the blend components in the membrane; ^(b)^ Average thicknesses; ^(c)^ Determined via TGA-FTIR coupling, a method described in [47]; ^(d)^ WU = water uptake at 25 and 90 °C; ^(e)^ λ = water molecules per SO_3_H group, calculated according to Equation (2) (see Section 2.3.1); ^(f)^ Determined via DSC; ^(g)^ Impedance determined at r.t. in 0.5 N H_2_SO_4_ solution using the Zahner EIS device; ^(h)^ Calculated from 1000 / Ω*cm.

## Data Availability

The data presented in this study are available on request from the corresponding author.

## References

[1] Babic U., Suermann M., Büchi F.N., Gubler L., Schmidt T.J. (2017). Critical Review—Identifying Critical Gaps for Polymer Electrolyte Water Electrolysis Development. J. Electrochem. Soc..

[2] Bessarabov D., Wang H., Li H., Zhao N. (2017). PEM Electrolysis for Hydrogen Production: Principles and Applications.

[3] Carmo M., Fritz D.L., Mergel J., Stolten D. (2013). A comprehensive review on PEM water electrolysis. Int. J. Hydrogen Energy.

[4] Grot W. (1972). Perfluorierte Ionenaustauscher-Membrane von hoher chemischer und thermischer Stabilität. Chem. Ing. Tech..

[5] Grot W. (1978). Use of Nafion Perfluorosulfonic Acid Products as Separators in Electrolytic Cells. Chem. Ing. Tech..

[6] Grot W.G. (1994). Perfluorinated ion exchange polymers and their use in research and industry. Macromol. Symp..

[7] Ito H., Maeda T., Nakano A., Takenaka H. (2011). Properties of Nafion membranes under PEM water electrolysis conditions. Int. J. Hydrogen Energy.

[8] Gagliardi G.G., Ibrahim A., Borello D., El-Kharouf A. (2020). Composite Polymers Development and Application for Polymer Electrolyte Membrane Technologies—A Review. Molecules.

[9] Shin D.W., Guiver M.D., Lee Y.M. (2017). Hydrocarbon-Based Polymer Electrolyte Membranes: Importance of Morphology on Ion Transport and Membrane Stability. Chem. Rev..

[10] Bartmann M., Kowalczik U. (1988). Zum Mechanismus der oxidativen Kupplung von Phenolen. Makromol. Chem. Phys..

[11] Xu T., Wu D., Seo S.-J., Woo J.-J., Wu L., Moon S.-H. (2012). Proton exchange composite membranes from blends of brominated and sulfonated poly(2,6-dimethyl-1,4-phenylene oxide). J. Appl. Polym. Sci..

[12] Noshay A., Robeson L.M. (1976). Sulfonated polysulfone. J. Appl. Polym. Sci..

[13] Xing P., Robertson G.P., Guiver M.D., Mikhailenko S.D., Wang K., Kaliaguine S. (2004). Synthesis and characterization of sulfonated poly(ether ether ketone) for proton exchange membranes. J. Membr. Sci..

[14] Cichon P.J., Krüger A.J., Krieg H.M., Bessarabov D., Aniol K., Kerres J. (2016). Sulfonated poly(arylene thioether phosphine oxide)s and poly(arylene ether phosphine oxide)s PBI-blend membranes and their performance in SO_2_ electrolysis. Int. J. Hydrogen Energy.

[15] Adamski M., Skalski T.J.G., Britton B., Peckham T.J., Metzler L., Holdcroft S. (2017). Highly Stable, Low Gas Cross-over, Proton-Conducting Phenylated Polyphenylenes. Angew. Chem..

[16] Schuster M., Kreuer K.-D., Andersen H.T., Maier J. (2007). Sulfonated Poly(phenylene sulfone) Polymers as Hydrolytically and Thermooxidatively Stable Proton Conducting Ionomers. Macromolecules.

[17] Büchi F.N., Gupta B., Haas O., Scherer G.G. (1995). Study of radiation-grafted FEP-G-polystyrene membranes as polymer electrolytes in fuel cells. Electrochim. Acta.

[18] Holmes T., Skalski T.J.G., Adamski M., Holdcroft S. (2019). Stability of Hydrocarbon Fuel Cell Membranes: Reaction of Hydroxyl Radicals with Sulfonated Phenylated Polyphenylenes. Chem. Mater..

[19] Siracusano S., Baglio V., Lufrano F., Staiti P., Aricò A.S. (2013). Electrochemical characterization of a PEM water electrolyzer based on a sulfonated polysulfone membrane. J. Membr. Sci..

[20] Chae K.-J., Kim K.-Y., Choi M.-J., Yang E., Kim I.S., Ren X., Lee M. (2014). Sulfonated polyether ether ketone (SPEEK)-based composite proton exchange membrane reinforced with nanofibers for microbial electrolysis cells. Chem. Eng. J..

[21] Jang I.-Y., Kweon O.-H., Kim K.-E., Hwang G.-J., Moon S.-B., Kang A.-S. (2008). Application of polysulfone (PSf)– and polyether ether ketone (PEEK)–tungstophosphoric acid (TPA) composite membranes for water electrolysis. J. Membr. Sci..

[22] Smith D.W., Oladoyinbo F.O., Mortimore W.A., Colquhoun H.M., Thomassen M.S., Ødegård A., Guillet N., Mayousse E., Klicpera T., Hayes W. (2013). A Microblock Ionomer in Proton Exchange Membrane Electrolysis for the Production of High Purity Hydrogen. Macromolecules.

[23] Schönberger F., Kerres J. (2007). Novel multiblock-co-ionomers as potential polymer electrolyte membrane materials. J. Polym. Sci. A Polym. Chem..

[24] Takamuku S., Jannasch P. (2012). Multiblock Copolymers Containing Highly Sulfonated Poly(arylene sulfone) Blocks for Proton Conducting Electrolyte Membranes. Macromolecules.

[25] Ghassemi H., McGrath J.E., Zawodzinski T.A. (2006). Multiblock sulfonated–fluorinated poly(arylene ether)s for a proton exchange membrane fuel cell. Polymer.

[26] Einsla M.L., Kim Y.S., Hawley M., Lee H.-S., McGrath J.E., Liu B., Guiver M.D., Pivovar B.S. (2008). Toward Im-proved Conductivity of Sulfonated Aromatic Proton Exchange Membranes at Low Relative Humidity. Chem. Mater..

[27] Roy A., Hickner M.A., Yu X., Li Y., Glass T.E., McGrath J.E. (2006). Influence of chemical composition and sequence length on the transport properties of proton exchange membranes. J. Polym. Sci. B Polym. Phys..

[28] Cui W., Kerres J., Eigenberger G. (1998). Development and characterization of ion-exchange polymer blend membranes. Sep. Purif. Technol..

[29] Kerres J.A. (2001). Development of ionomer membranes for fuel cells. J. Membr. Sci..

[30] Kerres J.A. (2005). Blended and Cross-Linked Ionomer Membranes for Application in Membrane Fuel Cells. Fuel Cells.

[31] Kerres J.A. (2015). Design Concepts for Aromatic Ionomers and Ionomer Membranes to be Applied to Fuel Cells and Electrolysis. Polym. Rev..

[32] Kerres J., Ullrich A., Meier F., Häring T. (1999). Synthesis and characterization of novel acid–base polymer blends for application in membrane fuel cells. Solid State Ion..

[33] Li Q.F., Rudbeck H.C., Chromik A., Jensen J.O., Pan C., Steenberg T., Calverley M., Bjerrum N.J., Kerres J. (2010). Properties, degradation and high temperature fuel cell test of different types of PBI and PBI blend membranes. J. Membr. Sci..

[34] Van Zyl A.J., Kerres J.A., Cui W., Junginger M. (1997). Application of new sulfonated ionomer membranes in the separation of pentene and pentane by facilitated transport. J. Membr. Sci..

[35] Gogel V., Jörissen L., Chromik A., Schönberger F., Lee J., Schäfer M., Krajinovic K., Kerres J. (2008). Ionomer Membrane and MEA Development for DMFC. Sep. Sci. Technol..

[36] Peach R., Krieg H.M., Krüger A.J., van der Westhuizen D., Bessarabov D., Kerres J. (2014). Comparison of ionically and ionical-covalently cross-linked polyaromatic membranes for SO_2_ electrolysis. Int. J. Hydrogen Energy.

[37] Chromik A., dos Santos A.R., Turek T., Kunz U., Häring T., Kerres J. (2015). Stability of acid-excess acid–base blend membranes in all-vanadium redox-flow batteries. J. Membr. Sci..

[38] Krajinovic K., Kaz T., Haering T., Gogel V., Kerres J. (2011). Highly Sulphonated Multiblock-co-polymers for Direct Methanol Fuel Cells. Fuel Cells.

[39] Kerres J., Schönberger F., Chromik A., Häring T., Li Q., Jensen J.O., Pan C., Noyé P., Bjerrum N.J. (2008). Partially Fluorinated Arylene Polyethers and Their Ternary Blend Membranes with PBI and H_3_PO_4_. Part I. Synthesis and Characterisation of Polymers and Binary Blend Membranes. Fuel Cells.

[40] Kerres J.A., Xing D., Schönberger F. (2006). Comparative investigation of novel PBI blend ionomer membranes from nonfluorinated and partially fluorinated poly arylene ethers. J. Polym. Sci. B Polym. Phys..

[41] Kerres J., Ullrich A., Hein M. (2001). Preparation and characterization of novel basic polysulfone polymers. J. Polym. Sci. A Polym. Chem..

[42] Titvinidze G., Wohlfarth A., Kreuer K.-D., Schuster M., Meyer W.H. (2014). Reinforcement of Highly Proton Conducting Multi-Block Copolymers by Online Crosslinking. Fuel Cells.

[43] Cho H., Krieg H.M., Kerres J.A. (2019). Performances of Anion-Exchange Blend Membranes on Vanadium Redox Flow Batteries. Membranes.

[44] Volkov V.I., Chernyak A.V., Golubenko D.V., Tverskoy V.A., Lochin G.A., Odjigaeva E.S., Yaroslavtsev A.B. (2020). Hydration and Diffusion of H^+^, Li^+^, Na^+^, Cs^+^ Ions in Cation-Exchange Membranes Based on Polyethylene- and Sulfonated-Grafted Polystyrene Studied by NMR Technique and Ionic Conductivity Measurements. Membranes.

[45] Bülbül E., Atanasov V., Mehlhorn M., Bürger M., Chromik A., Häring T., Kerres J. (2019). Highly phosphonated polypentafluorostyrene blended with polybenzimidazole: Application in vanadium redox flow battery. J. Membr. Sci..

[46] Cooper K.R. (2010). Progress Toward Accurate Through-Plane Ion Transport Resistance Measurement of Thin Solid Electrolytes. J. Electrochem. Soc..

[47] Kerres J., Ullrich A., Hein M., Gogel V., Friedrich K.A., Jörissen L. (2004). Cross-Linked Polyaryl Blend Membranes for Polymer Electrolyte Fuel Cells. Fuel Cells.

[48] Pei P., Wu Z., Li Y., Jia X., Chen D., Huang S. (2018). Improved methods to measure hydrogen crossover current in proton exchange membrane fuel cell. Appl. Energy.

[49] Trinke P., Haug P., Brauns J., Bensmann B., Hanke-Rauschenbach R., Turek T. (2018). Hydrogen Crossover in PEM and Alkaline Water Electrolysis: Mechanisms, Direct Comparison and Mitigation Strategies. J. Electrochem. Soc..

[50] Trinke P., Bensmann B., Reichstein S., Hanke-Rauschenbach R., Sundmacher K. (2016). Hydrogen Permeation in PEM Electrolyzer Cells Operated at Asymmetric Pressure Conditions. J. Electrochem. Soc..

[51] Mayerhöfer B., McLaughlin D., Böhm T., Hegelheimer M., Seeberger D., Thiele S. (2020). Bipolar Membrane Elec-trode Assemblies for Water Electrolysis. ACS Appl. Energy Mater..

[52] Bühler M., Hegge F., Holzapfel P., Bierling M., Suermann M., Vierrath S., Thiele S. (2019). Optimization of anodic porous transport electrodes for proton exchange membrane water electrolyzers. J. Mater. Chem. A.

[53] Krajinovic K. (2011). Synthese und Charakterisierung von Multiblock-co-Poly(aryl)-Ionomer(blend)membranen für den Einsatz in Direktmethanolbrennstoffzellen. Master’s Thesis.

[54] Liu B., Robertson G.P., Guiver M.D., Sun Y.-M., Liu Y.-L., Lai J.-Y., Mikhailenko S., Kaliaguine S. (2006). Sulfonated poly(aryl ether ether ketone ketone)s containing fluorinated moieties as proton exchange membrane materials. J. Polym. Sci. B Polym. Phys..

[55] Kerres J. (2006). Covalent-Ionically Cross-linked Poly(Etheretherketone)-Basic Polysulfone Blend Ionomer Mem-branes. Fuel Cells.

[56] Yu T.H., Sha Y., Liu W.-G., Merinov B.V., Shirvanian P., Goddard W.A. (2011). Mechanism for degradation of Nafion in PEM fuel cells from quantum mechanics calculations. J. Am. Chem. Soc..

[57] Freger V. (2009). Hydration of ionomers and Schroeder’s paradox in Nafion. J. Phys. Chem. B.

[58] Kreuer K.-D., Paddison S.J., Spohr E., Schuster M. (2004). Transport in proton conductors for fuel-cell applications: Simulations, elementary reactions, and phenomenology. Chem. Rev..

[59] Albert A., Barnett A.O., Thomassen M.S., Schmidt T.J., Gubler L. (2015). Radiation-Grafted Polymer Electrolyte Membranes for Water Electrolysis Cells: Evaluation of Key Membrane Properties. ACS Appl. Mater. Interfaces.

[60] Klose C., Saatkamp T., Münchinger A., Bohn L., Titvinidze G., Breitwieser M., Kreuer K.-D., Vierrath S. (2020). All-Hydrocarbon MEA for PEM Water Electrolysis Combining Low Hydrogen Crossover and High Efficiency. Adv. Energy Mater..

[61] Chae J.E., Lee S.Y., Baek S.Y., Song K.H., Park C.H., Kim H.-J., Lee K.-S. (2021). High-performance multiblock PEMs containing a highly acidic fluorinated-hydrophilic domain for water electrolysis. J. Membr. Sci..

[62] Zhang H., Li J., Tang H., Lin Y., Pan M. (2014). Hydrogen crossover through perfluorosulfonic acid membranes with variable side chains and its influence in fuel cell lifetime. Int. J. Hydrogen Energy.

[63] Wei G., Xu L., Huang C., Wang Y. (2010). SPE water electrolysis with SPEEK/PES blend membrane. Int. J. Hydrogen Energy.

